# Profiling of *Campylobacter jejuni* Proteome in Exponential and Stationary Phase of Growth

**DOI:** 10.3389/fmicb.2017.00913

**Published:** 2017-05-18

**Authors:** Hana Turonova, Nabila Haddad, Mathieu Hernould, Didier Chevret, Jarmila Pazlarova, Odile Tresse

**Affiliations:** ^1^Department of Biochemistry and Microbiology, Faculty of Food and Biochemical Technology, University of Chemistry and TechnologyPrague, Czechia; ^2^SECALIM UMR1014, Institut National de la Recherche AgronomiqueNantes, France; ^3^UMR1319 MICALIS, Plateforme d'Analyse Protéomique de Paris Sud-Ouest, Institut National de la Recherche AgronomiqueJouy-en-Josas, France

**Keywords:** foodborne pathogen, *Campylobacter jejuni*, growth, exponential phase, stationary phase, CosR, regulation

## Abstract

*Campylobacter jejuni* has been reported as a major cause of bacterial food-borne enteritides in developed countries during the last decade. Despite its fastidious growth requirements, including low level of oxygen and high level of CO_2_, this pathogen is able to persist in the environment without permanent loss of its viability and virulence. As *C. jejuni* is not able to multiply outside a host, the cells spend significant amount of time in stationary phase of growth. The entry into the stationary phase is often correlated to resistance to various stresses in bacteria. The switching between exponential and stationary phases is frequently mediated by the regulator sigma S (RpoS). However, this factor is absent in *C. jejuni* and molecular mechanisms responsible for transition of cells to the stationary phase remain elusive. In this work, proteomic profiles of cells from exponential and stationary phases were compared using 2-D electrophoresis (2DE) fingerprinting combined with mass spectrometry analysis and qRT-PCR. The identified proteins, whose expression differed between the two phases, are mostly involved in protein biosynthesis, carbon metabolism, stress response and motility. Altered expression was observed also in the pleiotropic regulator CosR that was over-expressed during stationary phase. A shift between transcript and protein level evolution of CosR throughout the growth of *C. jejuni* was observed using qRT-PCR and (2DE). From these data, we hypothesized that CosR could undergo a negative autoregulation in stationary phase. A consensus sequence resulting from promoter sequence alignment of genes potentially regulated by CosR, including its own upstream region, among *C. jejuni* strains is proposed. To verify experimentally the potential autoregulation of CosR at the DNA level, electrophoretic mobility shift assay was performed with DNA fragments of CosR promoter region and rCosR. Different migration pattern of the promoter fragments indicates the binding capacity of CosR, suggesting its auto-regulation potential.

## Introduction

*Campylobacter jejuni* is continuously reported as the main cause of bacterial food-borne infections in developed countries (EFSA and ECDC, 2015). The disease caused by this pathogen, namely campylobacteriosis, is triggered mainly by consumption of contaminated food or water, although a direct transmission from infected animals to human hosts can occasionally occur (Bronowski et al., 2014). It manifests as an acute inflammatory diarrhea with symptoms common to other bacterial enteritides—abdominal pain, fever and watery diarrhea often accompanied with the presence of blood and leukocytes in stool (Blaser and Engberg, 2008). In most cases, campylobacteriosis is self-limiting and does not require specific therapy, however severe autoimmune disorders, such as Guillain-Barré and Miller-Fisher syndromes (Salloway et al., 1996; Nachamkin, 2002), reactive arthritis (Pope et al., 2007), and inflammatory bowel disease (Rodriguez et al., 2006) may appear. These late-onset complications, together with long convalescence time and high occurrence of campylobacteriosis, are the reasons why the disease is ranked as an infection with one of the highest annual burden (Batz et al., 2012; Gibney et al., 2014; Mangen et al., 2015). Although various strategies have been adopted by member states of EU in order to decrease numbers of campylobacteriosis (Lin, 2009; Saxena et al., 2013), the prevalence of this disease remains very high. It is therefore necessary to identify genetic and environmental factors affecting the persistence of *C. jejuni* in the environment, in order to develop new methods mitigating the campylobacteriosis cases.

As a pathogen with fastidious growth requirements, *C. jejuni* puzzles scientists with its ability to withstand broad range of stresses encountered during its lifecycle. In other pathogens represented by *Escherichia coli, Salmonella* spp., *Shigella* spp., and *Vibrio* spp., general stress response is regulated by sigma factor RpoS that is also responsible for switching the growth to stationary phase (Duval et al., 2015). The entry to the stationary phase requires cooperation of the sigma factor and many other regulators (Llorens et al., 2010). When switching to the stationary phase, growth rate of cells dramatically decreases as a result of reduced protein synthesis. The overall role of RpoS is to ensure adaptation and resistance of the cells to challenging environments. It directly regulates 10% of *E. coli* genes (Weber et al., 2005) that play role in morphological changes of the cells, resistance to broad range of stresses (oxidative and osmotic stress, heat shock, pH changes, etc.), virulence, metabolic processes, and the GASP (growth advantage in stationary phase) phenotype (Martinez-Garcia et al., 2001; Raiger-Iustman and Ruiz, 2008).

Unlike other Gram-negative bacteria, *C. jejuni* lacks several stress response genes, including the sigma factor RpoS (Parkhill et al., 2000; Garenaux et al., 2008). Despite its small genome, this strictly microaerobic pathogen had to develop other mechanisms allowing its survival in stressful conditions, such as lack of nutrients in aquatic environments, or high concentration of oxygen when exposed to air or to an oxidative attack of macrophages. The molecular mechanisms responsible for its survival in food, persistence in the environment and virulence have not yet been fully understood. Similarly, no information concerning the transition of *C. jejuni* cells from exponential to stationary phase are available nowadays. Functional replacement of the sigma factor RpoS has not yet been described and the molecular mechanisms facilitating a cellular switch from exponential to stationary phase remain unknown. Therefore, in this work, proteomic profiles of the cells from exponential and early stationary phase of growth were compared using a 2-D electrophoresis (2DE) and quantitative real-time PCR (qRT-PCR), in order to contribute to better understanding of the molecular changes occurring during cellular transition from exponential to stationary phase.

## Materials and methods

### Bacterial strains and growth conditions

In this work, *C. jejuni* strain 81-176 isolated from a raw milk outbreak (Korlath et al., 1985) was used. Cells were resuscitated from a stock on Karmali agar plates (Oxoid) at 42°C for 48 hours (h) in stainless steel jars filled with gas mixture containing 5% O_2_, 10% CO_2_, and 85% N_2_ (microaerobic atmosphere). Grown cells were subcultured microaerobically in BHI (Merck) for 48 h at 42°C and 110 rpm, and then used for preparation of final suspension. The final suspension was cultivated at 42°C, with shaking at 110 rpm in microaerobic atmosphere for 7 h to harvest proteins in exponential phase and for 18 h for stationary phase. All experiments were performed in 3 biological replicates.

### Protein isolation purification

Proteins were extracted and purified as described previously (Bieche et al., 2012; Haddad et al., 2012). Briefly, cells were harvested by centrifugation at 7000 g, 4°C for 20 min and washed consecutively with 200 mM glycine (Sigma-Aldrich) and 100 mM Tris-HCl pH 7.0 (Sigma-Aldrich). Pellets were resuspended in 10 ml of a 10 mM Tris-HCl pH 7.0 and the cells were disrupted by series of 6 × 30 s sonication at 20 kHz with 6 min intervals on ice (Vibracell 72434, Bioblock Scientific). Cell debris was removed by two consecutive centrifugations at 10 000 g, 4°C for 20 min. Then, cytoplasmic proteins were separated from membrane fractions by ultracentrifugation at 188 000 g for 1 h at 4°C. The cytoplasmic protein fraction in the supernatant was treated with protease inhibitor cocktail tablets COMPLETE (Roche Diagnostics) and nuclease solution with final concentrations of 18 mg/ml RNAse and 9 mg/ml DNAse (Sigma-Aldrich). Protein samples were dialyzed using cellulose membrane tubing with a cut-off at 12 kDa (Sigma-Aldrich) against MilliQ water at 4°C with shaking for 3 days by refreshing the dialysis bath each day. Total protein concentration was determined using the Micro BCA™ Protein Assay Kit (Perbio-Science).

### Two-dimensional gel electrophoresis

A quantity of 100 μg of proteins isolated after 7 and 18 h of cultivation was concentrated using Concentrator 5301 (Eppendorf, France) at room temperature. Samples were mixed with rehydration buffer containing 6 M urea, 2 M thiourea, 4% CHAPS, 0,4% dithiothreitol (DTT), and 2% Bio-Lyse 3/10 Ampholyte (Bio-Rad) and few grains of bromophenol blue (BB). Proteins were then loaded into 17 cm IPG strips (pH 4-7, Bio-Rad) by active rehydration at 50 V for 12 h. After that, the isoelectric focusing (IEF) was performed using the Bio-Rad IEF program as follows: from 50 to 250 V for 3 h, from 250 to 6,000 V for 3 h and at 6,000 V until reaching 54,000 Vh. Strips were then equilibrated in migration buffer containing 6 M urea, 50 mM Tris-HCl (pH 8.8), 2% SDS, 30% glycerol, 2% DTT, 4% iodoacetamide, and few grains of BB. The second dimension was performed using SDS-PAGE with 12% acrylamide gels. The IPG strips were immobilized to the gel using 1% low-melting point agarose (Bio-Rad). The migration ran at 40 mA/gel using Protean II xi cell (Bio-Rad) at 14°C, until the bromophenol blue reached the base of the gels. Proteins in gels were finally silver stained and scanned with a GS-800 densitometer operated with the QuantityOne® software (Bio-Rad). Three independent experiments were performed, with two technical replicates for each of them.

### Statistical analysis and protein identification

The image analysis was performed using the Progenesis Samespots® software (NonLinear Dynamics). For the statistical analysis of the results, three independent experiments were performed, each with two technical replicates. Differences between the two conditions from the independent experiments and replicates were validated by Principal Component Analysis (PCA) and differences among matched spot intensities were statistically validated by ANOVA test at a 5% significance level. Lower and higher abundant proteins were taken into account only if the mean difference in spot intensities passed the threshold of 1.5. Predominant proteins whose amount differed significantly between the phases (*q*- and *p*-value ≤ 0.05, power ≥ 0.8, fold difference of spot intensities ≥ 1.5) were excised from BioSafe colloidal Coomassie blue (Bio-Rad) stained gels containing in total 700 μg of proteins. The excised proteins were analyzed after trypsin digestion with a mass spectrometer MALDI-TOF Voyager DE super STR (Applied Biosystems) at the PAPPSO platform of the INRA Center in Jouy-en-Josas (France). Gel plugs were first washed twice with 50% (v/v) acetonitrile, 25 mM ammonium carbonate in water, and then dried in a vacuum speed concentrator. Aliquot (10 μl) of a trypsin solution (Promega, 12.5 ng/μl in 50 mM NH_4_HCO_3_) was added to each sample and digestion was performed for 6 h at 37°C. A 1 μl aliquot of each supernatant was spotted directly onto the MALDI plate then dried at room temperature before adding 1 μl of the matrix solution (α-cyano-4-hydroxycinnamic acid, 3 mg/ml in 50% (v/v) acetonitrile 0.1% (v/v) trifluororacetic acid). Mass spectra were acquired on a Voyager-DE-STR (Applied Biosystems, Framingham, MA), equipped with a nitrogen laser (Laser Science, Franklin, MA). Tryptic autodigestion ion peaks, (M + H)^+^ = 2211.104 and 842.509 Da, were used for internal calibration of spectra. The peptide mass lists were used for protein identification in a *C. jejuni*, strain 81-176, amino acid sequence database (UniprotKB, 09.09.2010), using the Protein Prospector v 3.2.1 software set with the following parameters: mass tolerance <20 ppm, four peptides required to match, one missed cleavage, oxidized methionine and carbamidomethylated cysteine, respectively as possible and fixed modifications. Identification was evaluated with regard to sequence coverage, Mowse score, and compatibility between theoretical and experimental molecular weight and isoelectric point. Genes of proteins significantly over-expressed in one of the growth phases were then chosen for qRT-PCR experiments to verify their expression on translational level.

### RNA isolation and reverse transcription

Cells were harvested from cultures cultivated for 4, 7, 12, 16, 18, and 24 h by centrifugation at 3,300 g at 4°C for 6 min, pellets were homogenized with 1 ml of lysis solution Extract-All (Eurobio) according to manufacturer's guidelines. After that, 200 μl of chloroform were added to the suspension allowing separation of cell components. Water phase containing RNA was removed, precipitated with 500 μl of isopropanol and dissolved again in 50 μl of RNase-free water after washing in 75% cool ethanol. Samples were then treated with DNase I (Sigma-Aldrich) and purified using RNeasy Mini Kit (Qiagen) according to the manufacturer's instructions. Possible DNA contamination was detected by PCR detecting house-keeping gene *flaA* (Nachamkin et al., 1993). The integrity of RNA was verified using the Experion System (Bio-Rad). Its concentration and purity was measured using NanoDrop 2,000 (Thermo Scientific).

For reverse transcription 100 ng of RNA were mixed with 0.5 μl of 1 μM Random Hexamer Primer (Promega) and 4.5 μl of RNase-free water. This mixture was incubated 5 min at 70°C followed by 5 min on ice. After that 15 μl of Master Mix were added to mixture (5 μl of 5 × RT buffer, 1 μl 25 mM dNTP and 1 μl of M-MLV H^−^ reverse transcriptase; Promega) and it was incubated 10 min at room temperature, 50 min at 48°C and finally 15 min at 70°C to stop the reaction.

### Quantitative PCR (q-PCR)

Quantitative real-time PCR assay was performed using the AB7300 Realtime PCR system with SYBR Green-I Master Mix (Applied Biosystems). Primers (Eurobio) were designed with the on-line Primer3 software (www.simgene.com; Table 1) and their quality was checked both virtually, using FastPCR software, and by PCR. The efficiency of the primer pairs was calculated with the formula E = [10^(1/−s)^] × 100, where “s” is the slope of the standard curve. The efficiency of designed primers ranged from 101.27 to 105.38%.

**Table 1:** **Primers used in the experiments**.

**Experiment**	**Gene**	**Primer**	**5′to 3′**	**Product size (bp)**	**T_*a*_ (°C)**
qRT-PCR	*acnB*	AcnB Fw	AGCGGACTTGTAGCTTTTGC	98	60
		AcnB Rev	ACTCCAGCTTGCAATTCTCC		
	*betA*	BetA Fw	CACTGGGATGCGGATAATCT	105	59
		BetA Rev	AGCACAGCGATAAGCCAAAG		
	*cheW*	CheW Fw	GGTGAGACAAATGGAACTGGA	109	59
		CheW Rev	AAGTTTCAGGTGGTGGATCG		
	*cosR*	CosR Fw	TTTGAAAGCTGGAGCTGATG	100	59
		CosR Rev	GGTTCCGCCAAGTCTTAGTC		
	*dnaK*	DnaK Fw	CTTTCTTGGGCGAGAGTGTT	101	60
		DnaK Rev	TCCTGCTATCGTTCCTGCTT		
	*flaA*	FlaA Fw	AAAGCAGCAGAATCGCAAAT	110	58
		FlaA Rev	TTTGCTTGAGCCATTGCATA		
	*fumC*	FumC Fw	TGCGGTTGAGCAAGTAGAGA	118	59
		FumC Rev	TTGTAAATGCGTGCGTCCTA		
	*oorA*	OorA Fw	AGCGGTCCAGGAATTTCTTT	109	60
		OorA Rev	GAAGACCTGTCGAAGGACCA		
	*rbr*	Rbr Fw	GGCGAATCTATGGCAAGAAA	106	58
		Rbr Rev	TTTCATTTTCAGCCGCTTCT		
	*trxB*	TrxB Fw	CAGGGATAGGCTGTGCAGTT	146	60
		TrxB Rev	GGTTCCTTCGCATCCCTTAT		
EMSA		CosRpromF	TGGTATATTAGATTTCGAAAGAAG	374	ND
		CosRpromR	CTTCTATAACTAAAATTCTCAT		
		CosRintF	TTTTGATATTCTACTCGCAAGA	484	ND
		CosRintR	AAAAGAGACATCATAACCTTTCA		
		KatF2	CGTGCATCCCAGTGTTCTAT	337	ND
		KatR2	TTTTGCGCCTGCGCTTAATG		

The composition of q-PCR mix and amplification program were the same as described by Bieche et al. (2012) Amplification efficiency was determined from standard curve of serially diluted cDNA. The expression level of examined genes was calculated with AB 7,300 software against endogenous control gene *rrs* to obtain relative quantity.

### Purification of recombinant CosR

In order to produce sufficient amount of His_6_-tagged recombinant CosR (rCosR), restriction enzymes sites NdeI and BamHI were added to *cosR* sequence during its amplification using specific primers NtagCosR-NdeI (5'-GCACACATATGAGAATTTTAGTTATAGAAG-3′) and NtagCosR-BamHI (5′-TACAGGATCCAAGGTGCAAAATTGTTA-3′), to obtain NdeI-CosR-BamHI amplicons. These amplicons were inserted using NdeI and BamHI enzymes and subcloned into plasmid pET-15b (Novagen, France) to obtain a *N*-terminal His_6_ tagged protein under the control of T7 promoter. The resulting plasmid was transformed to competent *E. coli* expression strain BL21(DE3) (Novagen, France). Overexpression of rCosR was obtained from an overnight culture at 28°C in LB supplemented with 50 μg/ml of ampicillin, and 0.125 mM isopropyl-β-d-1-thiogalactopyranoside (IPTG). Cells were then harvested by centrifugation at 5,000 *g* for 10 min, washed with TE buffer (Sigma-Aldrich, France) and resuspended for 30 min in a lysis buffer containing 50 mM TrisHCl, pH 7.5, 300 mM NaCl, 5 mM β-mercaptoethanol and 1 mg/ml of lysozyme. The lysate was centrifuged and rCosR was purified using Ni-NTA agarose columns (Qiagen, France) according to the manufacturer's instructions. Columns were equilibrated using lysis buffer before protein binding and then washed sequentially with buffer W1 (lysis solution with 1% glycerol, 0.5% triton and 20 mM imidazole) and buffer W2 (lysis buffer with 20 mM imidazole). The recombinant protein was eluted using 400 mM imidazole and 6 M urea and purified by dialysis according to the manufacturer's instructions. Aliquots of rCosR were stored at −80°C.

### Bioinformatic analysis of the CosR DNA-binding box

A bioinformatic analysis was performed to specify the consensus sequence of the CosR DNA-binding box proposed by Hwang et al. (2011, 2012). For this purpose, sequences of 5′ 400-nt upstream regions of 7 genes previously reported as binding CosR were retrieved from the platform MicroScope (Microbial Genome Annotation & Analysis Platform; http://www.genoscope.cns.fr/agc/microscope/home/). To inspect the conservation of the CosR binding box, sequences of nine different fully sequenced *C. jejuni* strains were used in the analysis (Supplementary Table 1). Resulting consensus sequence logos were created using the WebLogo platform (http://weblogo.threeplusone.com; Crooks et al., 2004).

### Electrophoretic mobility shift assay (EMSA)

EMSA was carried out to identify putative CosR binding sites. DNA fragments covering promoter regulatory regions or gene internal regions of *cosR* and *katA* were PCR amplified using designed primers (Table 1). To demonstrate the DNA-binding properties of CosR, concentration range of purified recombinant protein CosR-His_6_ (1–10 μmol/l) were mixed with 2 μmol of purified PCR fragments in a total volume of 20 μl containing 20 mM Tris-HCl (pH 8.0), 150 mM NaCl, 5 mM MgCl_2_, 10 mM KCl, 10 μg/ml sheared salmon sperm DNA, and 10% glycerol. Sonicated salmon sperm DNA was applied as a nonspecific competitor. All samples were incubated at room temperature for 30 min following by the separation of the complexes using electrophoresis on a native 6% polyacrylamide gel in 0.5 × TBE.

## Results

Considering the lack of information concerning the transition of *C. jejuni* from exponential to stationary phase, we compared the proteomic profiles of cells harvested after 7 and 18 h of microaerobic cultivation at 42°C.

**Figure 1:**
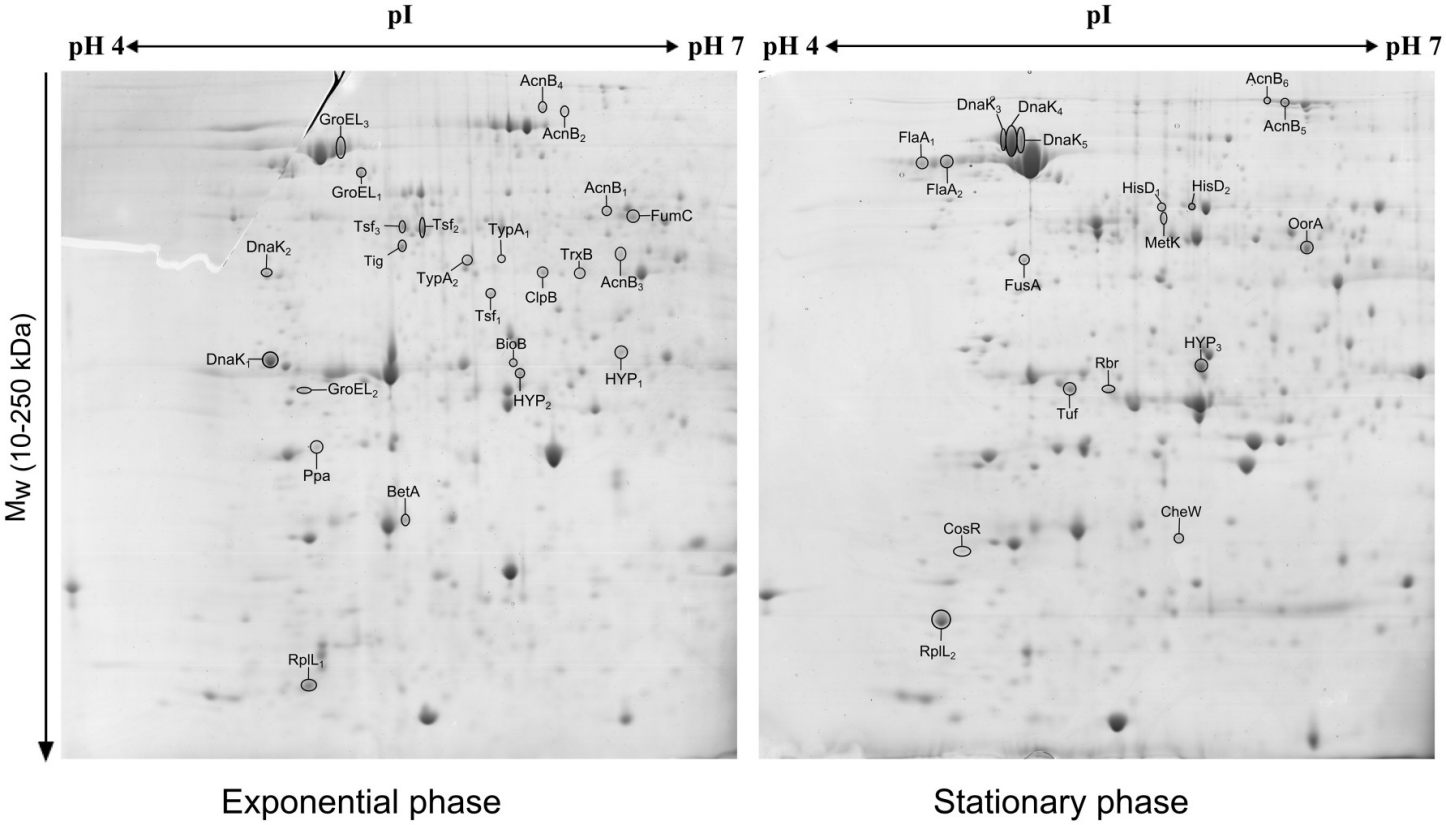
**2D electrophoretic profiles of *C. jejuni* proteins isolated after 7 h (exponential phase) and 16 h (early stationary phase) with highlighted overexpressed proteins**. A total of 700 μg of cytosolic proteins were separated on IPG strip (pH 4-7) followed by second separation by SDS-PAGE in 12% acrylamide gel stained by Coomassie blue.

The comparison of the 2-DE proteomic profiles revealed numerous differences between the two phases (Figure 1). In total, 42 spots were identified as 24 different proteins with 11 proteins more abundant in the exponential phase (17 spots), 10 proteins more abundant in the stationary phase (12 spots) and 3 proteins whose isoforms were overexpressed in both exponential and stationary phase (13 spots; Table 2). The identified proteins are involved mostly in metabolism (AcnB, BioB, FumC, HisD, MetK, and OorA), general and oxidative stress response (BetA, ClpB, CosR, DnaK, GroEL, Rbr, and TrxB), translation (FusA, RplL, Tig, Tsf, Tuf, and TypA), and motility (CheW and FlaA).

**Table 2:** **Identification of spots differently expressed in exponential phase (7 h) and early stationary phase (16 h) of *C. jejuni* growth**.

**Access No**.	**Protein**	**Respective gene**	**Theoretical Mw (kDa)/pI**	**Fold^a^**	***P*-value**	**MOWSE Score^b^**	**MP/PC^c^**
**METABOLISM**							
gi|121612675	Bifunctional aconitate hydratase 2/2-methylisocitrate dehydratase	*acnB*	92.69/5.89	−3.3	5.96E−04	3.78E + 04	12/20%
				−2.6	1.31E−03	4.37E + 04	11/16%
				−2.0	6.59E−05	2.04E + 02	5/8%
				−2.6	6.85E−05	8.95E + 05	13/18%
				+3.4	1.19E−05	1.09E + 06	15/22%
				+3.1	6.90E−04	8.25E + 07	18/26%
gi|121613366	Histidinol dehydrogenase	*hisD*	46.40/5.52	+4.2	4.03E−08	2.86E + 04	11/26%
				+2.8	1.18E−04	7.94E + 02	8/20%
gi|121612907	Biotin synthase	*bioB*	30.98/5.58	−3.3	4.85E−04	1.42E + 04	8/28%
gi|121613034	Fumarate hydratase	*fumC*	50.65/6.12	−2.2	4.16E−05	7.73E + 03	9/19%
gi|121612575	S-adenosylmethionine synthetase	*metK*	44.23/5.45	+3.4	3.50E−05	3.22E + 02	6/16%
gi|121613267	2-oxoglutarate-acceptor oxidoreductase subunit OorA	*oorA*	40.93/5.85	+3.0	3.22E−07	1.03E + 05	12/39%
**STRESS RESPONSE**							
gi|121612439	DNA-binding response regulator	*cosR*	25.54/5.27	+4.0	1.27E−05	1.47E + 03	8/37%
gi|121612363	Putative oxidoreductase	*betA*	63.66/8.75	−2.7	5.90E−06	1.35E + 02	8/12%
gi|121612692	Rubrerythrin	*rbr*	20.97/5.56	+5.3	1.51E−06	8.61E + 01	7/29%
gi|121612116	Pyridine nucleotide-disulphide oxidoreductase	*trxB*	33.78/5.93	−1.8	3.33E−06	1.63E + 05	12/43%
gi|121613623	ATP-dependent chaperone ClpB	*clpB*	95.55/5.47	−3.2	1.93E−04	2.60E + 04	15/13%
gi|121613084	Chaperone DnaK	*dnaK*	67.44/4.98	−4.3	6.65E−05	2.69E + 02	4/8%
				−2.5	3.17E−04	1.48E + 02	6/13%
				+6.8	3.20E−06	2.82E + 02	7/19%
				+3.5	4.84E−04	1.12E + 03	7/20%
				+3.4	9.69E−04	6.01E + 02	7/11%
gi|121612249	Chaperonin GroEL	*groEL*	57.97/5.02	−6.3	2.35E−06	1.61E + 04	14/32%
				−3.8	4.24E−05	3.52E + 01	6/7%
				−2.4	2.23E−03	1.70E + 04	13/34%
**MOTILITY**							
gi|121612156	Purine-binding chemotaxis protein	*cheW*	19.51/5.48	+3.9	1.18E−05	1.26E + 02	6/38%
gi|121612545	Flageline	*flaA*	59.54/5.61	+5.0	1.77E−04	8.27E + 04	9/19%
				+3.1	2.98E−03	4.21E + 02	7/15%
**TRANSLATION**							
gi|121612720	Elongation factor G	*fusA*	76.75/5.07	+4.3	3.05E−06	1.75E + 03	10/15%
gi|121613176	50S ribosomal protein	*rplL*	13.02/4.70	−3.8	1.01E−03	4.67E + 01	5/37%
				+2.8	4.53E−04	1.34E + 03	4/40%
gi|121612935	Trigger factor	*tig*	51.02/5.60	−2.5	1.06E−04	2.93E + 04	18/31%
gi|121612430	Elongation factor Tu	*tuf*	43.59/5.11	+3.1	4.40E−05	6.30E + 05	8/19%
gi|121612368	Elongation factor Ts	*tsf*	39.54/5.24	−4.6	7.18E−06	4.73E + 03	9/29%
				−3.2	2.51E−04	6.74E + 02	10/27%
				−2.1	5.03E−03	7.23E + 03	7/23%
gi|121612682	GTP-binding protein	*typA*	66.48/5.23	−2.5	4.81E−05	6.22E + 00	5/6%
				−1.9	7.77E−04	8.55E + 00	8/12%
**VARIOUS**							
gi|121613702	Inorganic pyrophosphatase	*ppa*	19.33/4.79	−1.8	6.24E−04	6.64E + 01	5/31%
gi|121613455	Putative zinc ribbon domain protein	CJJ81176_0729	27.74/5.60	−2.0	5.25E−03	2.96E + 03	8/27%
				−1.9	1.13E−02	1.19E + 04	14/50%
gi|121612896	Putative glutathione synthetase ATP-binding domain-like	CJJ81176_0107	38.79/5.23	+4.4	1.25E−07	1.72E + 02	8/15%

*The fold changes are normalized on the level of protein abundance detected in exponential phase*.

a *Positive values indicate a fold change for higher protein abundance in stationary phase. Negative values indicate a fold change for lower protein abundance in stationary phase*.

b Each protein was identified with a mass tolerance <20 ppm using at least four peptides

c MP/PC: Number of matched proteins/protein coverage

**Figure 2:**
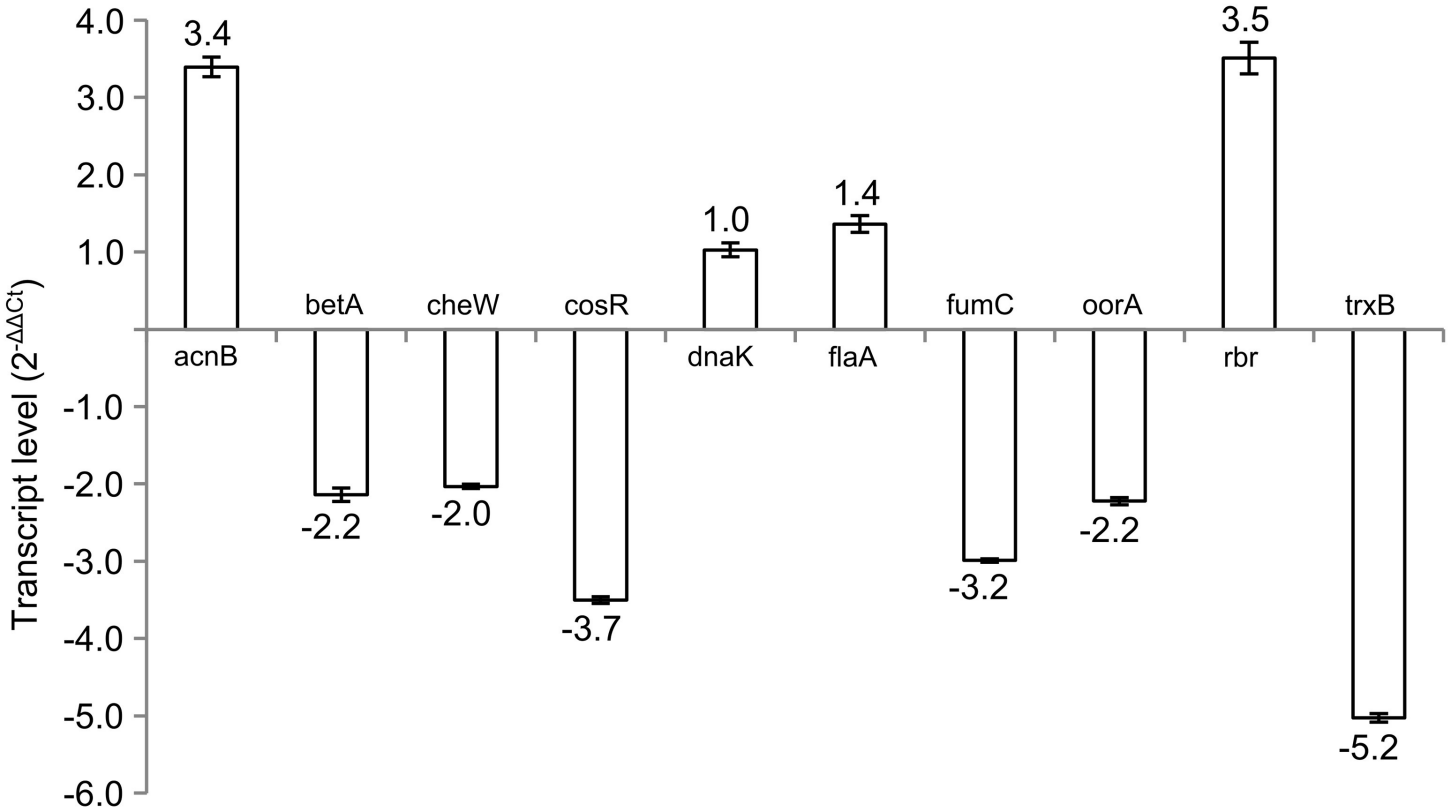
**Transcript levels of selected genes in early stationary phase (16 h) of *C. jejuni* growth normalized to the level of transcript from exponential phase (7 h)**.

Subsequently, 10 differently expressed proteins were selected for qRT-PCR analysis according to their potential to play role in the phase switch. The selected proteins participate in pathways important for the survival of *C. jejuni*, such as carbon metabolism (AcnB, FumC, and OorA), general and oxidative stress response (BetA, DnaK, Rbr, and TrxB), motility (FlaA, CheW) and gene expression regulation represented by the essential two-component regulator CosR. When comparing the level of the transcripts, 6 genes were more transcribed in exponential phase (*betA, cheW, cosR, fumC, oorA*, and *trxB*), while 3 genes were more transcribed in stationary phase (*acnB, flaA*, and *rbr*). There was no difference in the expression of *dnaK* (Figure 2). In most of the cases, the level of transcription corresponded with the level of protein expression. However, in three cases opposite trends were found between the 2-DE and qRT-PCR data, where the proteins were found to be overexpressed in the stationary phase, while the amount of mRNA was higher in the exponential phase. Those include OorA subunit of enzyme involved in carbon metabolism, CheW protein regulating chemotaxis, and regulator CosR.

**Figure 3:**
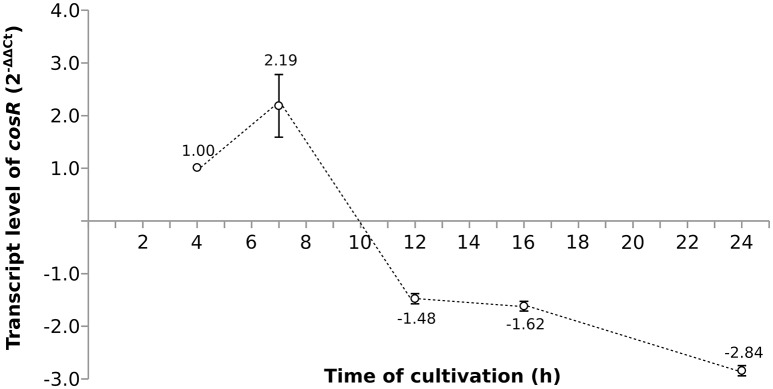
**The dynamics of *cosR* transcript level throughout the growth of *C. jejuni* normalized to the transcript level at 4 h**.

As the pleiotropic regulator CosR is essential for *C. jejuni* growth and regulates several pathways, CosR was selected for further analysis. To evaluate the changes of *cosR* transcript level throughout the growth of *C. jejuni*, total mRNA was extracted from the cells at different time-points of their cultivation and *cosR* specific transcript level was analyzed using qRT-PCR. The amount of *cosR* transcripts evolved during the growth of the strain 81-176 with an overall fold difference of 5.3. The highest level detected in cells was reached at 7 h of cultivation during the exponential phase and declined constantly afterwards in the stationary phase (Figure 3).

**Figure 4:**
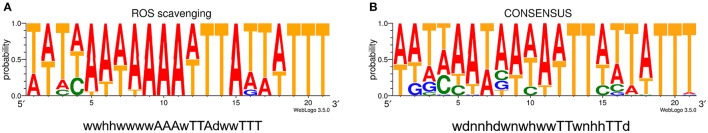
**Promoter sequence alignment. (A)** Consensus sequence logo from upstream sequence of the promoter region of genes responsible for ROS scavenging (*ahpC, cosR, katA* and *sodB*). **(B)** Consensus sequence logo of CosR-binding box of the upstream sequences of genes with CosR-binding capacity (*ahpC, cmeA, cosR, dps, katA, luxS*, and *sodB*). Both logos are based on sequences from 9 strains listed in Supplementary Table 1. D–A, T or G; H–A, T or C; N–A, T, C or G; W–A or T.

**Figure 5:**
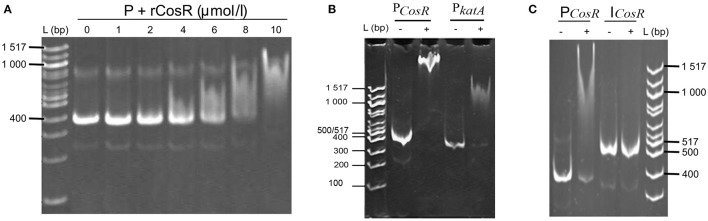
***In vitro*** **interactions of rCosR with promoter regions**. **(A)** Titration of a 374-bp *cosR* promoter DNA with rCosR. **(B)** Shift mobility of 374-bp *cosR* promoter DNA of *C. jejuni* 81–176 and the positive control using a 337-bp *katA* promoter DNA of *C. jejuni* NTCTC 11168 performed with 10 μmol/l rCosR. **(C)** Mobility of the promoter and internal DNA fragments of *cosR* with (+) or without (−) the addition of 10 μmol/l of the purified r*CosR* protein. L corresponds to 100 bp molecular weight ladder (NE Biolabs).

The comparison of the data obtained by qRT-PCR with the 4.0-fold protein increase in the early stationary phase led us to a hypothesis that CosR could undergo a negative autoregulation by binding to its own promoter region. The hypothesis was strengthened by identification of the CosR box described by Hwang et al. (2011, 2012) in *C. jejuni* 81-176 genome upstream from the *cosR* gene. Interestingly, the sequences of the CosR-binding boxes of 4 genes responsible for oxidative stress response are conserved among the analysed strains (Figure 4A) with the consensus sequence 5′-wwhhwwwwAAAwTTAdwwTTT-3′ which is similar to the one described by Hwang et al. (2012). To further refine the consensus sequence of the CosR box, 400-nt upstream sequences of the 6 genes previously described as binding CosR (*ahpC, cmeA dps, katA, luxS*, and *sodB*) by Hwang et al. (2011, 2012) and the one of CosR itself were compared among 9 fully sequenced *C. jejuni* strains (Supplementary Table 1). The refine CosR box resulted in the consensus sequence 5′-wdnnhdwnwhwwTTwnhhTTd-3′ (Figure 4B). In order to verify whether CosR can bind to its own promoter region, EMSA was performed (Figure 5). Regions containing the promoter region of *cosR* and an internal part of *cosR* gene were amplified and subjected to a concentration range of purified recombinant rCosR. Promoter region of *katA* from NCTC 11168 was used as a positive control validating the EMSA assays, as rCosR was previously shown to be able to bind to this DNA promoter region (Hwang et al., 2011). A mobility shift was observed when the DNA fragments of *cosR* and *katA* promoters were used, while no motility shift was observed for the internal DNA region of *cosR*, indicating the potential capacity of CosR to bind to its own promoter.

## Discussion

*Campylobacter jejuni* is repeatedly reported as the major cause of food-borne bacterial enteritides worldwide, causing severe economic and sociological burden. This generally stress-sensitive bacterium, that supposedly cannot multiply outside a host, is able to survive harsh conditions encountered in the environment (Whiley et al., 2013). Despite the concern of this pathogen, relatively little is known about the molecular mechanisms responsible for its ability to persist in meat processing facilities, on carcasses and in the food environment. Enhanced bacterial resistance to various stresses is often connected to the transition of the cells into different phases of growth. For example in the case of *E. coli*, cells in stationary phase were found to be more resistant to high hydrostatic pressure, mild heat, oxidative and osmotic stress, and chlorine disinfectants (Kaur et al., 1998; Benito et al., 1999; Cherchi and Gu, 2011). However, *Campylobacter* cells were reported as generally more resistant in exponential phase of growth, with dynamic emergence of resistant subpopulations in the stationary phase (Kelly et al., 2001, 2003). Molecular mechanisms responsible for the transition between the growth phases have not yet been fully described. Unlike other bacteria, *C. jejuni* lacks sigma factor RpoS regulating the switch from one phase to another (Parkhill et al., 2000). According to several authors, post-transcriptional regulator CsrA could contribute to modulation of gene expression in *C. jejuni* in response to growth phase. Indeed, in *E. coli* and *S. enterica* Thyphimurium, CsrA participates in the regulation of genes inducible in stationary phase (Romeo, 1998; Lawhon et al., 2003). Similar data were recently reported also for *C. jejuni* 81-176 where CsrA strongly influences expression of proteins in stationary phase and might therefore compensate the lack of RpoS (Fields et al., 2016). Another potential regulator that might participate in the transition could be the stringent response regulator *spoT*, as it was previously reported as important for survival of cells in stationary phase (Gaynor et al., 2005). As regulations during phase transition in *C. jejuni* remain unexplained at the molecular level, proteomic fingerprinting of cells isolated from exponential and stationary phase were compared in this work, to identify the main protein changes occurring between the two main growth phases of *C. jejuni* 81-176.

The first group of identified proteins is involved in cellular stress response. This group contains mostly proteins participating in oxidative stress and protein folding. Interestingly, there is no clear pattern of phase-depending overexpression of these proteins. However, two out of three chaperons were overexpressed in exponential phase, suggesting higher abundance of misfolded and aggregated proteins in the exponential phase, probably caused by the request of functional *de novo* proteins for rapid biosynthesis of cellular material during cell multiplication. The overexpression of stress-related proteins is not surprising, as toxic metabolites accumulate during growth in batch cultivation. The presence of toxic metabolites together with starvation occurring in the stationary phase could be the reasons why the cells overexpressed proteins involved in the stress response. The overexpression of stress response proteins, reported previously by Wright et al. (2009), could speed up the adaptation process of *C. jejuni* when changing the environment. Such adaptation would also require adjustment of metabolism reflecting the changes in the availability of nutrients during the cultivation of the cells. Indeed, in this work, we also observed altered expression of several proteins playing role in *C. jejuni* metabolism, mostly of those involved in the citric acid cycle (TCA). Asaccharolytic bacterium *C. jejuni* uses TCA as the main carbon source through gluconeogenesis (Velayudhan and Kelly, 2002). Unlike other bacteria, the TCA cycle of *C. jejuni* involves enzymes containing oxygen-sensitive Fe-S clusters, such as Oor enzymes (Atack and Kelly, 2009) that are rapidly inactivated after exposure to ambient air (Kendall et al., 2014). Higher abundance of OorA observed in the stationary phase could be explained by successful detoxification of ROS with the help of TrxB and BetA which are among the overexpressed protein in the exponential phase.

Another group of proteins with altered expression between the two growth phases is involved in protein biosynthesis. The proteins overexpressed in the exponential phase were mostly elongation factors responsible for correct synthesis of new peptides. The proteins whose abundance is higher in the stationary phase play role in binding of tRNA to ribosomes and their translocation from A to P site. These results suggest high importance of protein biosynthesis in *C. jejuni* regardless of the physiological state of the cells, although they are in contrast with the decline of ribosomal proteins in the stationary phase reported previously (Wright et al., 2009).

Interestingly, the cells in stationary phase overexpressed proteins involved in motility (flagellar protein FlaA) and chemotaxis (regulator CheW). Similar overexpression was already reported both for cells in stationary phase (Wosten et al., 2004; Wright et al., 2009; Fields et al., 2016) and in biofilms (Kalmokoff et al., 2006). The overexpression of FlaA in stationary phase is mediated by CsrA (Fields et al., 2016) and could be explained by multifunctionality of *C. jejuni* flagella. Besides their role in motility, flagellum apparatus works as a type III secretion system (Konkel et al., 2004) and it was found to be crucial for *C. jejuni* adhesion and biofilm formation (Svensson et al., 2014). In our case the protein regulating chemotaxis was also overexpressed, suggesting that the chemotaxis-driven motility is enhanced in stationary phase, facilitating the migration of cells toward nutrient-rich environment.

The transcript analysis revealed that for most of the tested genes, the level of the transcription was correlated to the variation in protein abundance, indicating a regulation of the corresponding proteins at the transcriptional level. However, in three cases (CosR, CheW, and OorA) the level of transcripts was higher in the exponential phase while the protein abundance was significantly lower as compared to the amount in the stationary phase. Such difference could be explained by post-trancriptional or post-translation modifications (Maier et al., 2009). The translation can be delayed when Shine-Dalgarno sequence of mRNA sequence is not completely complementary to the rRNA sequence (Maier et al., 2009) or when the mRNA undergoes conformational change, e.g., by binding of small metabolites (Nahvi et al., 2004). Another reason can be the involvement of short regulatory sRNA that is influencing stability of mRNA and translation process by targeting or blocking mRNA binding to ribosomes (Maier et al., 2009). In addition, overall number of available ribosomes and half-life of translated proteins also play role in final mRNA-protein ratio. Among the proteins presenting an opposite trend between transcript level and protein amount, only CosR is identified as a DNA regulator. This recently discovered orphan two component system (TCS), whose sequence is conserved among *Campylobacter* and *Helicobacter* species, affects transcription of 93 different genes, including 17 genes essential for *C. jejuni* viability (Hwang et al., 2012). It is involved in the regulation pathway of ROS detoxification (Hwang et al., 2011), efflux pump expression (Hwang et al., 2012), and also influences biofilm formation and maturation of *C. jejuni* (Oh and Jeon, 2014; Turonova et al., 2015). The *cosR* gene is present in all *C. jejuni* strains (Gundogdu et al., 2016). It is an essential gene for *C. jejuni* with the absence of a consensus catalytic aspartic acid replaced by an asparagine. It shares 60% amino acid identity with Hp1043, a response-regulator element of TCS from *Helicobacter pylori* (Beier and Frank, 2000; Schar et al., 2005; Stahl and Stintzi, 2011). The interchange of *C. jejuni cosR* with homologous gene from *H. pylori* does not impair its functionality, indicating a probable conserved function of the gene throughout strains and closely related bacterial genera (Muller et al., 2007). As proteomics analyses using time-point profiling are restricted, dynamics of transcript levels and protein abundance of CosR were explored.

The results indicated a maximum transcribed amount of *cosR* after 7 h of cultivation in exponential phase followed by a decline up to 24 h of cultivation, while the abundance of the protein was 4-times higher at the early stationary phase. The transient upregulation of the transcript level and the protein amount during the growth led us to a hypothesis that CosR could potentially undergo negative autoregulation. Negative autoregulation, or negative autogenous regulation, is one of the three common motifs used for bacterial regulation of transcription (Shen-Orr et al., 2002), where transcription factor negatively regulates its own transcription (Savageau, 1974). This type of autoregulation has been previously detected in *E. coli*, where it concerns over 40% of all transcription factors, including global regulators Crp, Hns, Ihf, and Lrp (Shen-Orr et al., 2002). The kinetics of transcription is generally slow, faster kinetics can be achieved by shortening the lifetime of a product by degradation, which is inconvenient due to metabolic cost (Rosenfeld et al., 2002). As it was proven both mathematically and experimentally, negative autoregulation speeds up the transcription response (Rosenfeld et al., 2002; Shen-Orr et al., 2002). However, autoregulation requires active binding of the transcription factor to the promoter region of the operon. Previous studies of binding of CosR–DNA sequences in upstream regions of some genes revealed the presence of a specific CosR-binding box (Hwang et al., 2011, 2012). The resulting consensus sequence of the box was estimated from sequences of 5 genes in *C. jejuni* NCTC 11168. The authors considered some of the nucleotides to be identical although they were not present in all cases (4/5 for positions 3, 9, 11, 14, 20, and 21). The only identical nucleotides present in all 5 examined regions were on positions 5, 10, 15, and 19. Our analysis show lower level of similarity of the CosR boxes. When we compare the sequences of genes responsible for ROS scavenging (*ahpC, cosR, katA*, and *sodB*), the CosR-binding box is conserved and similar to the one reported by Hwang et al. (2012). This consensus sequence was refined using 63 sequences from upstream promoter regions of all genes previously described to bind CosR and the own promoter region of CosR. The resulting consensus sequence indicated a well conserved motif at the end of the consensus sequence which might be valuable for further analyses of the CosR regulon. To detect whether CosR could potentially bind to its own promoter, DNA shift mobility analysis was used. The data indicated a shift mobility of CosR in presence of its own promoter region sequence, which indicates that CosR is also able to bind its own promoter region. Therefore, it might have a potential to undergo a negative autoregulation. The autoregulation of CosR could be also influenced by the post-transcriptional regulator CsrA, which was recently reported to affect CosR expression (Fields et al., 2016). However, due to broad spectrum of genes under CrsA regulation it is not clear whether the observed difference in CosR expression is caused by direct or indirect regulation. Further analyses are therefore required to decipher a potential role of CsrA in cosR expression. Taken together, these data suggest a potential binding of CosR to its own promoter, although additional analyses are required to confirm the *in vivo* protein-DNA complexation. Future analyses should focus on this potential complexation of CosR with its own promoter and its impact on gene expression.

In summary, our proteomic data show that the transition from exponential to stationary phase is accompanied with numerous changes in the gene expression. The main functional groups of differently expressed proteins involve those playing role in metabolism, stress response, translation, and motility, suggesting active adaptation of the cells to the environment. Our transcriptomic and proteomic analyses also pinpointed the dynamics of the CosR expression, leading to the hypothesis of negative autoregulation of this pleiotropic regulator. Altogether, we outlined mechanisms that *C. jejuni* could use during the growth.

## Author contributions

OT conceived the study. OT, NH, MH, and HT designed the experiments. HT and MH performed the experiments, DC performed the protein identification. HT and MH analyzed the results and HT prepared the draft manuscript. NH, JP, and OT contributed to the manuscript.

### Conflict of interest statement

The authors declare that the research was conducted in the absence of any commercial or financial relationships that could be construed as a potential conflict of interest. The reviewer BM and handling Editor declared their shared affiliation, and the handling Editor states that the process nevertheless met the standards of a fair and objective review.

## Supplementary material

Supplementary Table 1 **CosR-binding box sequences of genes used in the bioinformatic analysis**.
